# The effect of salt substitution on frequency and severity of headache: results from the SSaSS cluster-randomised controlled trial of 20,995 participants

**DOI:** 10.1038/s41430-024-01419-7

**Published:** 2024-02-24

**Authors:** Faraidoon Haghdoost, Sonali R. Gnanenthiran, Sana Shan, Prachi Kaistha, Liping Huang, Maoyi Tian, Yishu Liu, Xuejun Yin, Xinyi Zhang, Zhixin Hao, Yangfeng Wu, Gian Luca Di Tanna, Bruce Neal, Anthony Rodgers

**Affiliations:** 1 The George Institute for Global Health, University of New South Wales (UNSW), Sydney, New South Wales Australia; 2 Concord Repatriation General Hospital, Concord West, New South Wales Australia; 3 The George Institute India, Hyderabad, India; 4 School of Public Health, Harbin Medical University, Harbin, China; 5 The George Institute for Global Health, Peking University Health Science Center, Beijing, China; 6 School of Public Health, Imperial College London, London, UK

**Keywords:** Preclinical research, Cerebrovascular disorders, Hypertension, Risk factors, Translational research

## Abstract

**Background:**

Headache is one of the most common neurological symptoms. Headache disorders are associated with a high global burden of disease. Prior studies indicate that short-to-medium term sodium reduction reduces headache symptom. This study evaluated the effects of long-term reduced-sodium, added-potassium salt on headache frequency and severity in rural China.

**Methods:**

The Salt substitute and stroke study (SSaSS) was an open-label cluster-randomised trial in rural China designed to evaluate the effect of salt substitution on mortality and cardiovascular events. Participants included adults with a history of prior stroke and those aged ≥60 years with uncontrolled high blood pressure (BP). Villages were randomly assigned in a 1:1 ratio either to intervention with salt substitute (75% sodium chloride and 25% potassium chloride by mass) or to control with continued use of regular salt (100% sodium chloride). In this pre-specified analysis, between-group differences in headache frequency and severity were evaluated. The study was registered with ClinicalTrials.gov (identifier number: NCT02092090).

**Results:**

A total of 20,995 participants were included in the trial (mean age 64.3 years, 51% female, mean follow-up 4.7 years). At final follow-up at the end of the study, headache outcome data including frequency and severity of headaches was available for 16,486 (98%) of 16,823 living participants. Overall, 4454/16,486 (27%) individuals reported having headache: 27.4% in the intervention group (2301/8386) vs 26.6% in the control group (2153/8100) (RR 1.04, 95% CI: 0.93, 1.16, *p* = 0.48). There was no difference in headache severity between intervention and control groups (*p* = 0.90).

**Conclusion:**

Long term salt substitution did not reduce the frequency or severity of headaches in this population.

## Introduction

Headache is the most common neurological presentation and is associated with a high burden of disability and substantial global impact [1, 2]. According to the Global Burden of Disease Study, in 2016, approximately three billion people globally had either tension-type headaches or migraine, the two primary headache disorders that are the third and sixth most common health disorders, respectively [3]. Management of headache is both pharmacological and non-pharmacological, including dietary approaches [4]. Potential dietary strategies include weight reduction, especially in obese patients, low caloric and ketogenic diets, decreased omega-6, increased omega-3 fatty acids and avoiding some dietary triggers such as alcohol, chocolate, and caffeine overuse [4, 5]. A limited number of studies based principally on the results of short-term trials of sodium reduction have reported the impact of salt reduction on reducing headache [6, 7]. However, the long-term effects of salt substitution on headache are unknown. We therefore conducted a pre-specified analysis of the Salt Substitute and Stroke Study (SSaSS) to determine whether five years of dietary sodium reduction using a potassium-enriched salt substitute reduced headache frequency and severity compared to regular salt intake in rural China.

## Methods

### Study design

The details of the SSaSS trial have been reported elsewhere [8]. In brief, SSaSS was an open-label large-scale, cluster-randomised, controlled trial conducted in 600 villages across five provinces of Northern China (Hebei, Liaoning, Ningxia, Shanxi, and Shaanxi). The provinces were selected because of the high prevalence of hypertension and stroke. The villages were randomised to receive salt substitute or a continued usual diet with regular salt. The SSaSS study showed a protective effect of sodium reduction using a salt substitute on the risk of cardiovascular events and mortality [9]. The headache outcome was added during the study protocol amendment (protocol date: 15/5/2019, ethics approval date: 27/6/2019), and the analysis plan was finalized before data lock [9].

The ethics committees of Peking University Health Science Centre, China (IRB00001052-13069), and the University of Sydney, Australia (2013/888), approved the study. An independent Data Monitoring Committee oversaw the study, and informed consent was obtained from all the participants.

### Participants

Adults with a high risk of developing a stroke were included in this study. Approximately 35 individuals from each of the 600 villages were recruited using the following inclusion criteria: (a) prior history of stroke (regardless of underlying aetiology) and/or (b) age ≥60 years with uncontrolled high blood pressure (BP) defined as systolic BP ≥ 140 mmHg after two measurements in people using BP-lowering drugs or systolic BP ≥ 160 mmHg after two measurements in individuals receiving no medication for high BP. In addition, the participant or a household member had to own a phone and provide another person’s number as an emergency contact.

Exclusion criteria were: (a) using potassium-sparing diuretics or potassium supplements; (b) diagnosis of severe renal impairment; (c) any concern about using salt substitute; (d) life expectancy less than six months from the trial commencement; (e) eating most meals outside of the home. Informed consent was obtained from every participant, and group consent was obtained from local county Bureaus of Health leadership.

### Randomisation and masking

Villages were randomly assigned in a 1:1 ratio to the intervention group or the control group. Randomisation was stratified according to county, using a central computerised process. Random assignment of the villages was performed after all the participants in the province had been recruited and all baseline survey data had been collected. Based on the nature of the study, the intervention’s delivery could not be masked.

### Procedures

There were two groups in the study: the Intervention group that received salt substitute and the control group that continued the usual diet.

Intervention: Participants and their households in villages randomised to the intervention used the salt substitute sufficient for all daily cooking and seasoning needs instead of the regular salt. Salt substitution is a form of salt that has 75% sodium chloride and 25% potassium chloride by mass compared to regular salt which is 100% sodium chloride. Each household received about 20 g of salt substitute per person per day (maximum of 20 kg per year per household) free of charge. Participants were advised to use the salt substitute instead of regular salt and use it less frequently than their previous salt usage habits. In addition, participants received general health advice regarding stroke prevention and healthy behaviours.

Control: Villages in the control group continued the usual diet with regular salt (100% sodium chloride) and were advised to use salt less frequently than their previous salt usage habits. This group also received general health advice for stroke prevention and healthy behaviours at the beginning of the study, like the intervention group.

After obtaining informed consent, baseline data including age, gender, body mass index (BMI), any history of smoking, history of stroke, hypertension, diabetes Mellitus, and use of anti-hypertensive drugs were collected via interview, thereafter a physical examination which included systolic BP (SBP), and diastolic BP (DBP), assessments was performed. The village doctor was responsible for distributing the salt substitute but was not involved in any outcome collection or assessments. The trained outcome assessors oversaw evaluations in a similar way in all the included villages and were instructed not to ask about the randomisation status. Outcome assessment was performed in person if the participants were available in the village on the visit day; otherwise, it was undertaken via phone call or by rescheduling to another day.

### Outcomes

Headache frequency and severity outcomes were evaluated at final follow-up only (five years) by recording the number of days in the past month that a participant experienced a headache (headache frequency)—(a) 0 days, (b) 1–10 days, (c) 11–20 days, (d) >20 days, or (e) unknown. Participants reporting headache were asked whether the severity of headaches was mild, moderate, severe or unknown. Given this was a pragmatic, large scale trial, no details about headache were collected at baseline and no details of headache type were recorded.

### Statistical analysis

Primary analyses were performed according to the intention-to-treat principle. Statistical analyses were performed using SAS software, version 9·4 (SAS Institute). Binary outcome of headache incidence (0 days vs ≥1 days) was analysed using hierarchical log-binomial regression model: results presented as rate ratios, 95% confidence intervals, and *p* values. Ordered categorical outcomes of headache frequency and headache severity were analysed using hierarchical ordered logistic regression model: results presented as odds ratios, 95% confidence intervals, and *p* values. All hierarchical models adjusted for clustering at the village level. For continuous outcomes including pulse pressure, blood pressure reduction, change in sodium excretion and potassium excretion: mean difference estimates, confidence intervals and *p* values were obtained from an analysis of covariance that allowed for clustering.

Subgroup analysis was performed to calculate the risk of having headache compared to no headache in the last month for groups defined by baseline age, BMI, SBP and DBP, sex, education, and history of disorders such as stroke, hypertension, and diabetes.

The study was registered with ClinicalTrials.gov in March 2014 with the identifier number NCT02092090.

### Role of the funding source

The trial was funded by the National Health and Medical Research Council of Australia (NHMRC) Project Grant (APP1049417), NHMRC Program Grant (APP1052555), and NHMRC Centre for Research Excellence Grant (APP1117300).

## Results

**Fig. 1:**
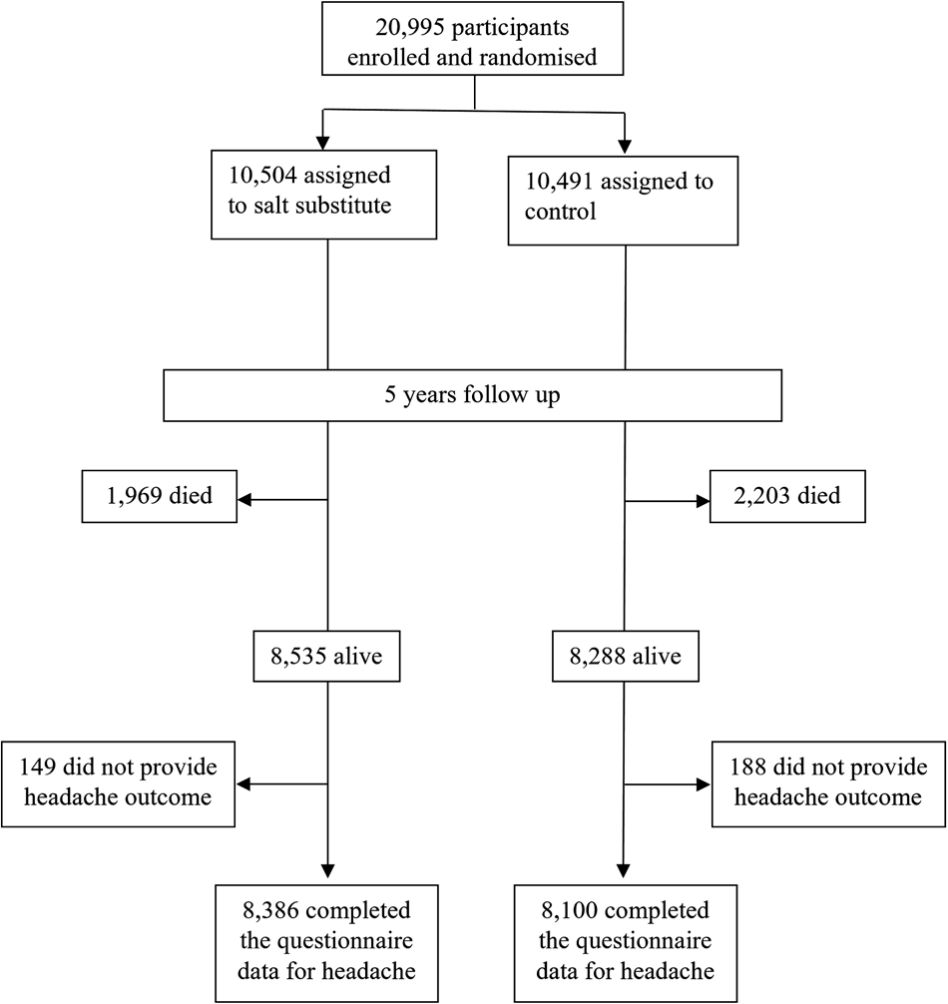
Trial profile. Enrollment, randomisation, and Follow-up processes.

A total of 20,995 participants were recruited from April 2014 to January 2015: 10,504 participants in the intervention group and 10,491 individuals in the control group. Of all randomised participants, 16,823 were alive at final follow-up. A total of 16,486 participants answered headache-related questions at the end of the study and were included in the current analysis (79% of all randomised and 98% of all alive) (Fig. 1). There were outcome data for all 600 of the villages, with between 12 and 35 participants providing data in each. The mean follow-up duration was 4.7 years. The baseline clinical characteristics of the participants are shown in Table 1. The mean age (±SD) of participants was 64.3 ± 8.1 years, and 51% were female. At 60 months follow-up visit, mean (±SD) SBP was 142.9 ± 20.5 and 146.5 ± 21.1 and DBP was 84.6 ± 11.7 and 85.5 ± 12.1 in the intervention and control groups, respectively. During the course of the trial, mean SBP was 3·4 (95% CI: 1.8, 4.9, *p* ≤ 0.001) mmHg lower and DBP was 0.8 (95% CI: –0.1, 1.7, *p* = 0.08) mmHg lower in the intervention compared to the control group. Standard deviation of BP change, as a measure of BP variability, was 27.4 vs 27.8 mmHg for SBP and 15.9 vs 16.3 mmHg for DBP in intervention and control. Pulse pressure at final visit was 58.3 ± 15.8 in intervention and 61.0 ± 16.3 in control. The mean difference in pulse pressure at 60 months was –2.6 mmHg (95% CI: –3.5, –1.6, *p* ≤ 0.0001). During the trial, mean 24-h urinary sodium level decreased by 15.3 (95% CI: 8.3, 39.0, *p* = 0.204) mmol and potassium level increased by 24.9 (95%CI: 17.5, 32.4, *p* ≤ 0.001) mmol in the intervention compared to the control group.

**Table 1:** Participants characteristics at baseline.

Characteristics	Intervention (*n* = 8386)	Control (*n* = 8100)	Total (*n* = 16,486)	Total randomized (*n* = 20,995)
Age (years)	64.2 ± 8.1	64.4 ± 8.1	64.3 ± 8.1	65.4 ± 8.5
Female- %	51.7%	51.3%	51.5%	49.5%
BMI (kg/m^2^)	24.9 ± 3.5	25.1 ± 3.6	25.0 ± 3.6	24.8 ± 3.6
BP (mmHg)				
- SBP	153.2 ± 23.2	153.8 ± 23.4	153.5 ± 23.3	154.0 ± 23.5
- DBP	88.9 ± 13.8	89.3 ± 13.8	89.1 ± 13.8	89.2 ± 14.0
Smoking –%				
- Current smokers	18.4%	19.0%	18.7%	18.8%
- Ever smoked cigarettes	32.1%	33.3%	32.7%	33.5%
History of prior stroke with diagnosis made in hospital - %	72.0%	70.4%	71.2%	72.7%
History of hypertension – %	88.7%	88.4%	88.5%	88.4%
History of diabetes mellitus –%	10.0%	10.0%	10.0%	10.6%
AHTDs – %				
- Beta blockers	5.1%	6.8%	5.9%	5.7%
- ACEi or ARB	23.7%	23.7%	23.7%	23.0%
- Calcium antagonist	44.0%	42.1%	43.1%	42.0%
- Diuretic	11.8%	11.5%	11.6%	11.3%

Data are reported as mean ± SD or number (%).

*BMI* body mass index, *BP* blood pressure, *SBP* systolic blood pressure, *DBP* diastolic blood pressure, *AHTDs* anti-hypertensive drugs, *ACEi* angiotensin-converting enzyme inhibitor, *ARB* angiotensin receptor blocker.

Of 16,486 participants alive at final follow-up, 4454 (27.0%) reported headache in the last month. In the intervention and control groups, 2301 (27.4%) and 2153 (26.6%) reported having headache (≥1 day) in the last month respectively (RR = 1.04, 95% CI: 0.93, 1.16, *p* = 0.48). There was also no between-group difference in the frequency of headache: for intervention vs control 1697 (20.2%) and 1558 (19.2%) reported 1–10 headache days per month, 306 (3.6%) and 297 (3.7%) reported 10–20 headache days per month, and 298 (3.6%) and 298 (3.7%) reported more than 20 headache days per month, respectively (*p* = 0.57).

**Fig. 2:**
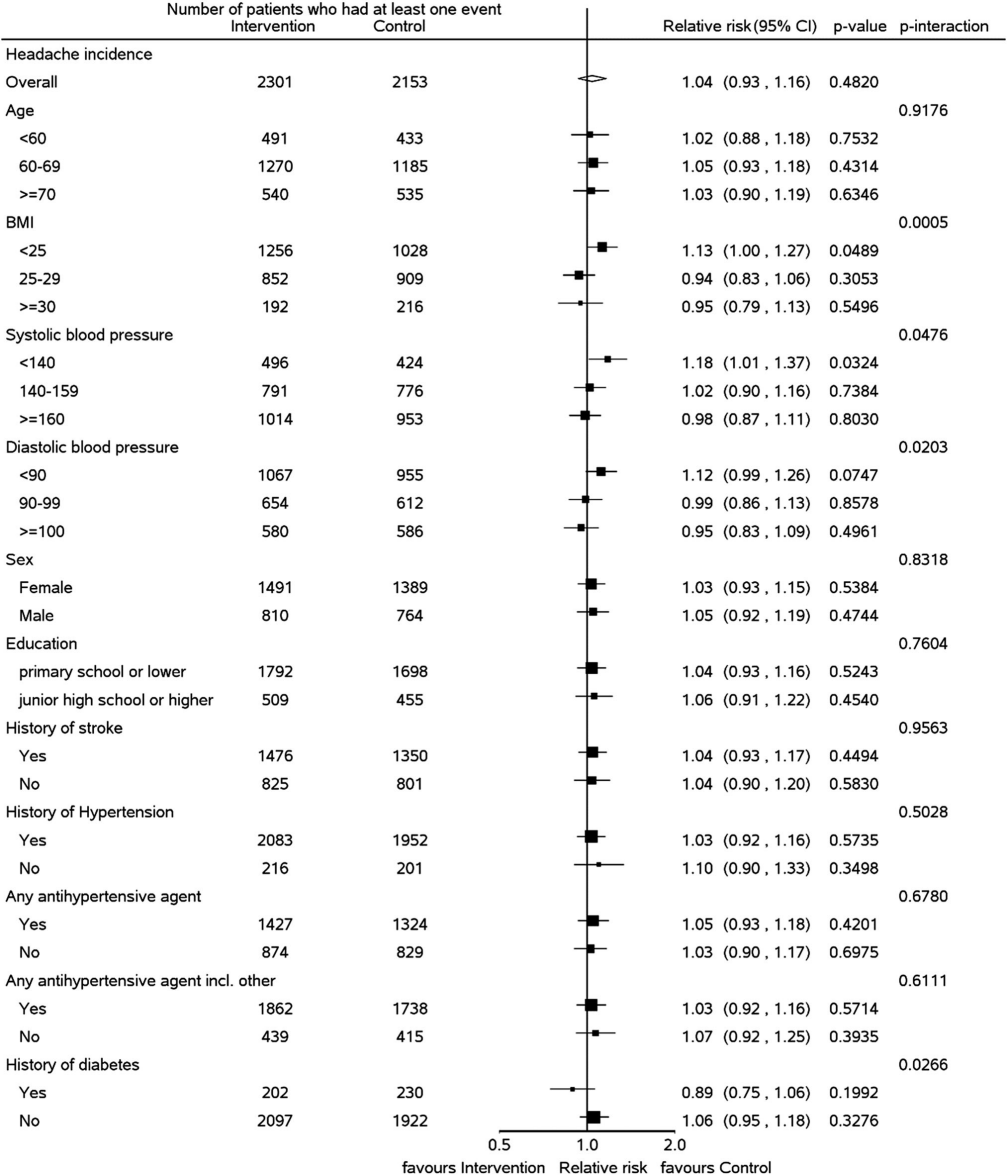
Subgoup analysis. Relative risk of any headache vs no headache in the last month in subgroups based on different variables is reported.

Further analysis on subgroups defined by sex, age, BMI, BP, education, and history of hypertension, diabetes, and stroke, is reported in Fig. 2. Of the 11 subgroups assessed, there was some evidence of interaction by BMI (*p* value for interaction <0.001) and SBP and DBP level, with *p* values of interaction of 0.05 and 0.02, respectively.

There was no evidence of change in headache severity. Out of participants with headache, 61.9% and 61.4% reported mild headache, 23.3% and 23.9% reported moderate headache and 7.9% and 7.4% reported severe headache in intervention and control groups respectively (*p* = 0.90).

## Discussion

These results indicate that salt substitution does not change headache frequency in participants with hypertension or cerebrovascular disease. Even though the study had large numbers and almost one-third of the participants reported having headaches, there were no significant differences between salt substitution and control groups regarding the frequency or severity of headaches.

The current study has several limitations. Although headache was a prespecified secondary outcome, the study was primarily conducted to evaluate the effect of salt substitution on cardiovascular outcomes. This study was a pragmatic trial in which headache was included as a supplementary outcome. Although the level of detailed outcome data for individuals was limited, the large sample size provided good power to detect average effects across the two groups. Additionally, even though data were collected at only one timepoint for each individual, they were gathered from different individuals over many months. This approach should, on average, mitigate the effects of factors such as seasonal variability. Neither baseline data on headache nor follow-up data on type of headache were collected as the study was highly pragmatic, with limited resources available. Therefore, an evaluation of the intervention on headache subtypes meeting international classification of headache disorders [10] was not possible. Finally, ~20% of participants died during follow-up and headache data prior to their deaths was unavailable. However, data for alive people was 98% complete and all villages (clusters) were involved in the analysis. Hence, this is the largest trial to assess the effect of long-term salt substitution on headache, and a high-level data completeness was achieved.

These results should be considered in the context of other relevant evidence. The data available is heterogeneous, with some studies suggesting benefits and others showing no reduction in headache with salt substitution. There have not been any clinical trials designed to assess the effect of salt reduction on headache as a primary outcome. Two prior studies performed a secondary analysis to evaluate the impact of dietary sodium on blood pressure. In the TONE study (*n* = 975 elderly individuals, median follow-up 29 months), Chen et al. demonstrated decreased risk of headache in the sodium reduction group (hazard ratio 0.59, 95% CI = 0.40, 0.88) compared to the control group [6, 11]. Similarly, Amer et al. reported that dietary sodium reduction was associated with a lower risk of headache after 90 days follow up in 390 participants in the Dietary Approaches to Stop Hypertension (DASH) study [7]. Another study on 681 hypertensive participants showed that after a mean follow-up of about 28 months, participants in the reduced sodium intake group reported fewer headaches than the control group [12].

In contrast, other trials have reported similar results to our findings and did not find any association between dietary sodium reduction and headache [13–16]. The majority of these studies have had only small sample sizes (from 16 to 132 participants) and short follow-ups (1–4 weeks), except for our current large-scale trial and Li et al. study [15] (*n* = 2555, 18-month follow-up). The latter similarly reported no significant difference in the headache report rate between sodium reduction and control groups (28.9% vs 28.3% had headache at the end of the study, respectively).

There are several possible reasons for this difference. The lack of significant BP reduction in the salt substitution trials may potentially be relevant, since previous trials of BP lowering drugs indicated the degree of headache reduction was proportional to the degree of DBP reduction [17, 18] and Mendelian randomisation analyses also suggest the importance of diastolic blood pressure for headache [19]. Other factors may also be relevant. Webb et al. reported that BP-lowering medications that increase BP variability reduce headache [18], whereas the SSaSS intervention did not affect BP variability. Another factor may be the reduced pulse pressure seen with the SSaSS intervention, which in the Nord-Trøndelag Health Study (HUNT) cohort study was associated with increased headache frequency (both migraine and non-migraine headaches) [20]. Finally, the modest size of changes in sodium and/or potassium intake may be relevant, beyond their role in affecting BP. Based on 24-h urinary excretion, SSaSS intervention participants had only a 15 mmol reduction in urine sodium and 25 mmol increase in urine potassium excretion compared to control participants [9]. In contrast, sodium reduction studies found a larger reduction in 24-h urinary sodium excretion (40–75 mmol) and no significant change in potassium excretion [6, 7, 12].

Our results showed a negative association between salt intake level and headache rate in individuals with lower BMI. Although this was based on subgroup analysis and making any conclusion should be done with caution, it is like the report by Pogoda et al. [21] in which the negative association in women was limited to those with lower BMI [21]. The association of increased dietary sodium intake and less headache in migraine patients is suggested in those with withdrawal from processed food. Less processed food causes a withdrawal headache due to less salt and, therefore increase in salt intake helps relieve the headache [22].

Salt reduction may not directly impact headache in individuals aged ≥60 years with a high risk of developing cardiovascular outcomes. However, based on our review, reducing DBP might be associated with a lower headache rate. The protective effect of salt reduction on cardiovascular events, especially hypertension, and the potential association of headache and BP suggests that salt reduction may benefit people with headaches. However, this effect may be dose dependent and requires further studies.

## Conclusion

Among people who had a history of stroke or hypertension, salt substitution did not affect headache frequency or severity. The reasons for this null effect in contrast to the benefits seen with sodium restriction are unclear, but may include the lack of effect on DBP. Further research is required to determine whether different types or intensity of salt substitution could reduce headache, while also reducing BP which mediates the benefits for CVD events seen in the SSaSS trial.

## Data Availability

Data access to SSaSS project can be requested from Professor Bruce Neal, via the email: bneal@georgeinstitute.org.au. Once the data access is granted, data can be accessed via a secured environment hosted by The George Institute for Global Health, China, in line with China’s Cyber Security Law.
